# Intramuscular orbital schwannoma: case report and review of the literature

**DOI:** 10.3389/fopht.2025.1586465

**Published:** 2025-05-23

**Authors:** Hammam A. Alotaibi, Firas Madani, Rawan N. Althaqib, Hamad M. AlSulaiman

**Affiliations:** ^1^ Oculoplastics & Orbit Division, King Khaled Eye Specialist Hospital, Riyadh, Saudi Arabia; ^2^ Department of Ophthalmology, Prince Sultan Military Medical City, Riyadh, Saudi Arabia; ^3^ Department of Ophthalmology, Faculty of Medicine, King Abdulaziz University, Jeddah, Saudi Arabia

**Keywords:** orbital schwannoma, diplopia, magnetic resonance, inferior rectus muscle, proptosis, case report

## Abstract

Orbital schwannomas are benign tumors that arise from Schwann cells in the peripheral nerves in the orbit. They typically present after the second decade of life given their slow growth and rarely before then. Diagnosis is based on clinical course and specific imaging modalities; however, the definitive diagnosis is by lesion biopsy. Surgical removal is typically curative. Herein we present the case of an 8-year-old boy with proptosis and diplopia where he exhibited the clinical findings of an orbital mass, however, the characteristic picture of orbital schwannoma was observed on imaging yet found within the inferior rectus muscle, a rare finding indeed.

## Introduction

Schwannomas, as the name suggests, are benign tumors that originate from Schwann cells in the peripheral nerves. They are rare findings, accounting for less than 1% of all orbital tumors. Given their slow growth, they are observed in patients between the ages of 20 and 70 years and rarely before. Pinpointing the origin of schwannoma is difficult, given the complex nature of orbital structures. However, they most commonly originate from branches of oculomotor, trochlear, trigeminal, and abducens nerves (1, 2).

When small, these tumors are asymptomatic and do not cause any noticeable effect on globe position, extraocular motion, or nerve function. However, as they grow, such symptoms develop and progressively worsen over time, mainly when located along the orbital apex (3).

Diagnosis of orbital schwannoma is made through imaging techniques such as Computerized tomography (CT) scans and magnetic resonance imaging (MRI) scans with or without contrast. However, the definitive diagnostic tests are biopsy (incisional vs. excisional) and immunohistochemistry.

The principal value of a CT scan is to identify the location/extension of the lesion, whether contained entirely in the orbit or causing bony remodeling/erosion and affecting structures beyond the orbit into the sinuses, for example, and by doing so, assists in differentiating lesions with clinically similar presentation (4). MRI is the method of choice in diagnosing schwannomas, given its high sensitivity. The lesion typically has low intensity on T-1 weighted images and high intensity on T-2 weighted images (5).

Surgical excision is curative and has a low recurrence rate. On histological examination, schwannoma will have one of two characteristic cellular morphological patterns denoted Antoni type-a and Antoni type-b. Immunohistochemistry shows a sensitivity of schwannomas to S-100 and vimentin stains, which are proteins prevalent in Schwann cells (6).

Herein, we describe a case of an 8-year-old with proptosis due to an orbital schwannoma found within an unusual structure, the inferior rectus muscle.

## Case description

An 8-year-old boy presented to the emergency department complaining of headache, progressive proptosis, and intermittent hyperdeviation of the right eye for one year. Upon presentation, his visual acuity was 20/20; there was no headache nor any signs of elevated intracranial pressure, such as nausea and vomiting. Examination revealed hypertropia and proptosis of the right eye with limited extraocular movement of -1 in supraduction and -2 in infraduction and a 2 mm difference between eyes on exophthalmometer. There was no afferent pupillary defect and no visual field defect on confrontation test.

**Figure 1:**
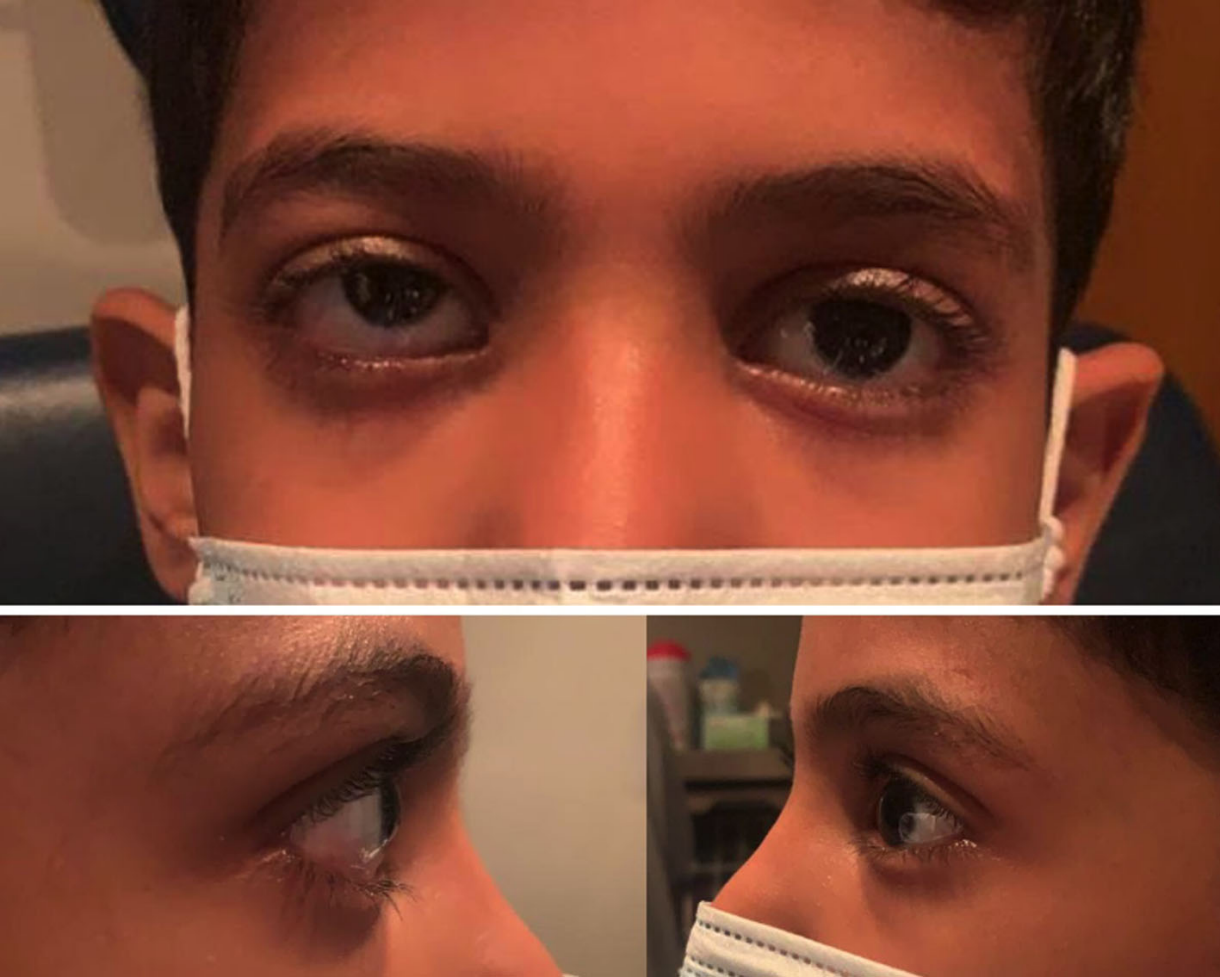
At presentation: right eye hypertropia and proptosis compared to the left eye.

Examination of the right eye shows a grossly obvious elevation of the right lower lid. Upon palpation, a firm mass too deep for size assessment was noted. However, it was non-tender and did not bruit on auscultation, as seen in (**Figure 1**).

The anterior and posterior segment examinations were unremarkable, showing quiet phakic eyes with clear corneas, a deep anterior chamber, a flat retina with good foveal reflex, and normal, healthy discs in both eyes.

## Diagnostic assessment

A computerized tomography scan (CT scan) is indicated for this case due to a suspicious area of bone erosion. Moreover, a biopsy was scheduled along with the CT scan.

**Figure 2:**
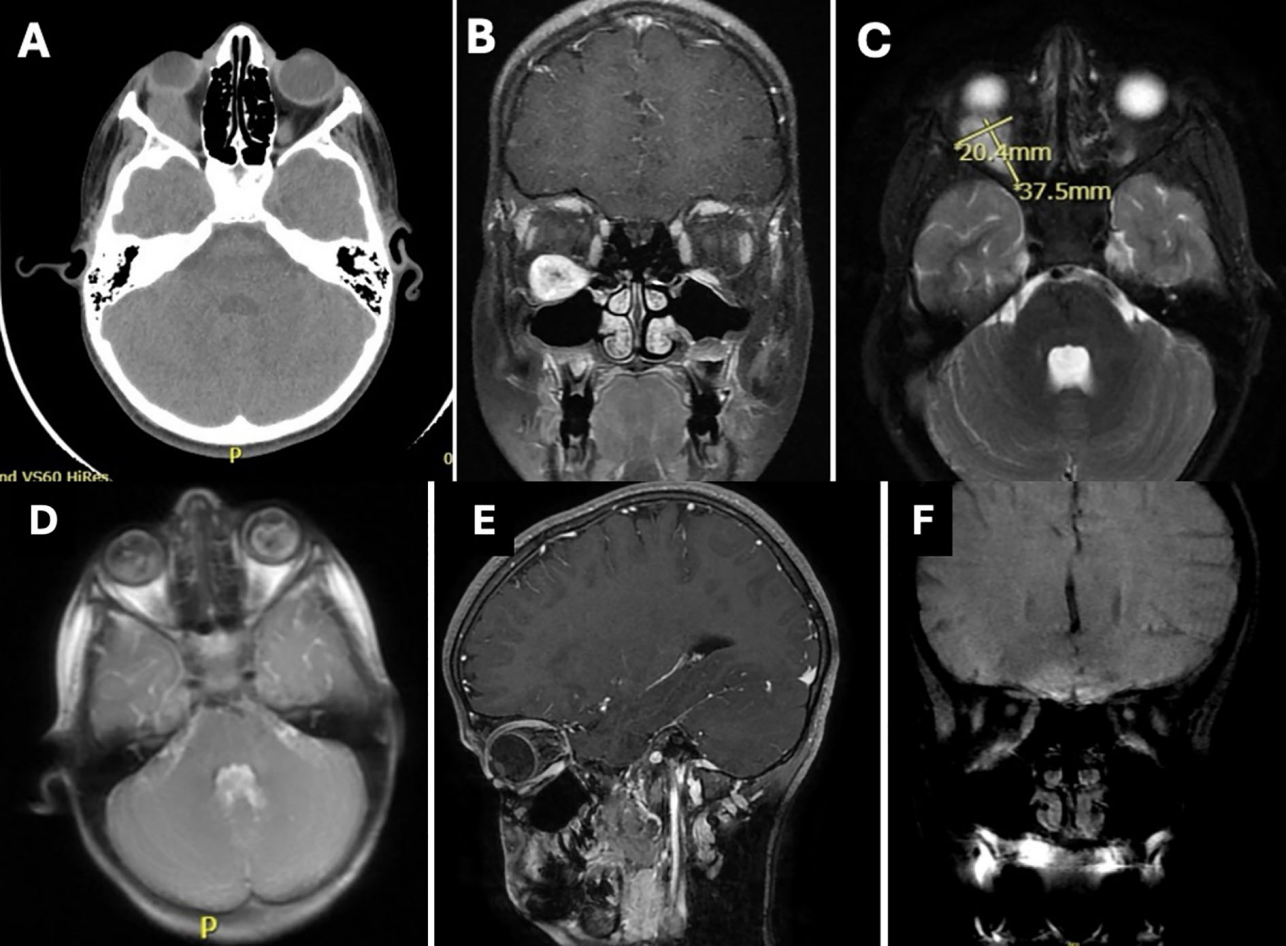
Imaging at presentation demonstrating location and size of the lesion. **(A)** Axial section CT brain and orbit. **(B)** Coronal section MRI upon presentation showing mass along the inferior rectus muscle. **(C)** T2 MRI axial section with lesion dimensions noted. Imaging following resection of the lesion at different times. **(D)** Axial section MRI, **(E)** sagittal section MRI; both after surgery showing complete resection of the lesion. **(F)** coronal section MRI after two years demonstrating no recurrence and return of the normal orbital anatomy.

The CT scan shows a solid-appearing inferior orbital mass lesion that is seen at the anatomical location of the right inferior rectus muscle belly, causing significant scalloping and thinning of the adjacent part of the right inferior orbital wall. The muscle cannot be separated from the adjacent muscle along its insertion. No other extraocular muscle group involvement of the same pattern. No fat density. As seen in (**Figure 2A**).

Magnetic resonance imaging (MRI) was ordered for the patient. An intraconal mass with a spindle shape configuration was noted with a well-circumscribed outline and measuring 37.5 x 20.4 mm. The central part of the mass shows a pericystic component. The lesion is in line with the inferior rectus muscle and cannot be separated from the adjacent part of the muscle. Other extraocular muscles and orbital structures, including the optic nerve, were clear of the lesion. there was no sinus extension (**Figures 2B, C**).

Upon excision, complete resection was achieved by blunt dissection, preserving the muscle and removing the mass whole. The orbital mass consisted of round, spongy, whitish-tanned tissues. Upon dissection, the cut surface was yellowing white and smooth at the periphery and had central hemorrhagic cavities.

**Figure 3:**
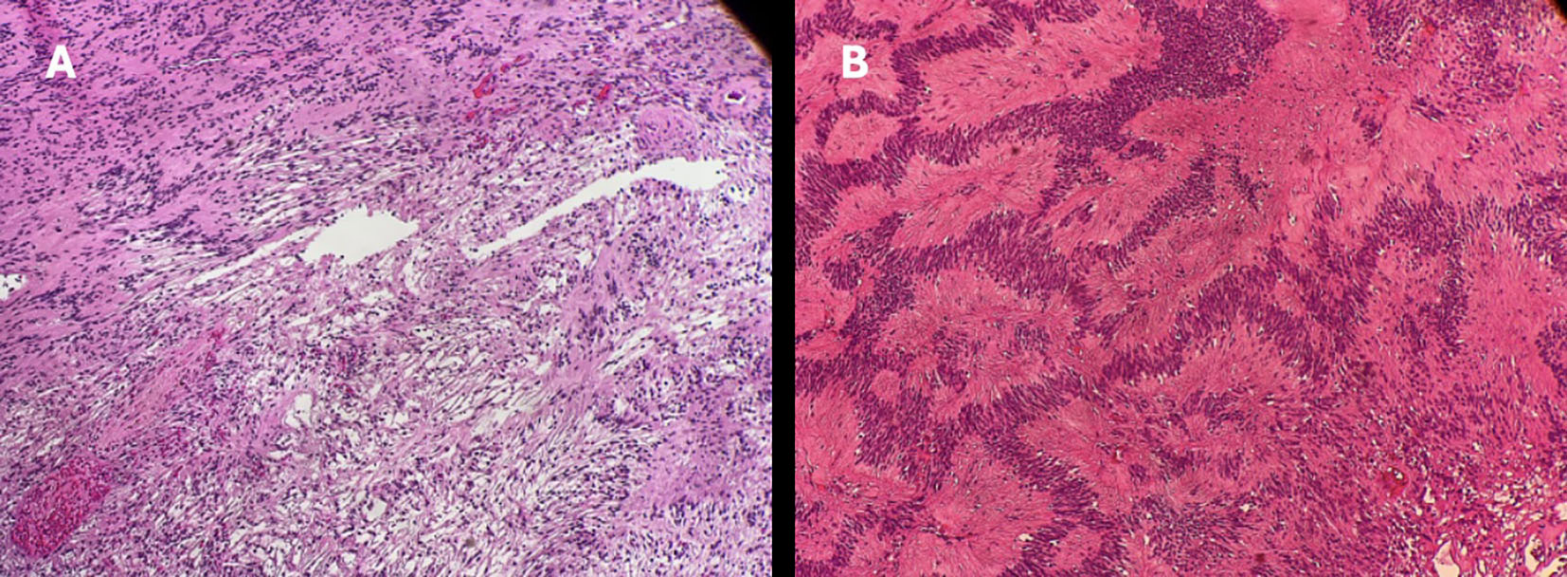
H&E stain of lesion biopsy demonstrating: **(A)** antoni A and antoni B patterns. **(B)** Verocay bodies. Both typical findings of schwannomas.

Histologically, the section contained a soft tissue tumor surrounded by a Perineural fibrous capsule. The tumor consists of spindle cell tumor palisading forming Antoni A and Antoni B arranged with a pattern with numerous Verocay bodies. Findings were consistent with right orbital schwannoma (**Figure 3**). No genetic testing was done for this patient given that it is not available in our institution.

At last visit, 2 years following the surgery, the patient was stable, had quiet eyes, minimal scarring of the palpebral conjunctiva of the right lower lid, clear cornea, deep anterior chamber and normal healthy disc. The patient had no diplopia in primary gaze and lower gaze but complained of minimal diplopia in upper gaze.

An MRI of the orbit following the surgical resection (**Figures 2D, E**) and at last follow up two years later (**Figure 2F**) demonstrating no recurrence and return of normal orbital anatomy.

## Discussion

Schwannomas account for 1% of all orbital tumors and develop almost exclusively between the ages of 20 and 70 years, making its development/detection in the pediatric population quite rare indeed (1, 2). Given the nature of this benign tumor, where it develops clear and encapsulated borders, complete surgical excision is usually successfully curative with a minimal recurrence rate. Diagnosis is based on characteristic imaging (CT scans and MRI) and histological/immunohistochemical findings (5, 6).

A CT scan is a useful tool in diagnosing schwannomas, given its utility in distinguishing whether the lesion is limited to the orbit, extending to the sinuses, or damaging/eroding the surrounding bony structures. However, schwannomas share the same radiographic findings as other lesions, such as cavernous hemangiomas, so further imaging and histologic testing are necessary for diagnosis.

The characteristics on a CT scan show a retrobulbar, smooth, ovoid solitary mass in the orbit with the long axis along the direction of the optic nerve. The lesion is usually isodense compared to the brain. Schwannomas that undergo cavitation over time will still present as an isodense lesion on CT; however, MRI can delineate these characteristics with more detail.

Consequently, MRI is considered the imaging method of choice to diagnose suspected schwannomas due to its high sensitivity especially when used with contrast. Schwannomas of the orbit appear on MRI as low-intensity signal on T1-weighted images and high-intensity signal on T2-weighted images, which can be homogeneously or heterogeneously enhanced (5).

Schwannomas are diagnosed histologically by the presence of one of two patterns in cellular morphology: first, Antoni type A, which appears as close-packed spindle cells with a fusiform nucleus and eosinophilic cytoplasm. Nuclei in type-a palisade form packed spindle-like structures in a “picket fence” pattern, which interdigitates cytoplasmic processes, forming a pattern known as Verocay bodies. Second, the Antoni type-b pattern demonstrates haphazard cellular distribution with distinct cytoplasmic margins. Immunohistochemical analysis of schwannoma cells shows positive staining for S-100 and vimentin, both of which are known to be present in Schwann cells (6).

Apart from the case we report, there are six other published cases of intramuscular orbital schwannoma in the literature, but only one was as young as the one presented herein as summarized in **Table 1**.

**Table 1:** Summary of published cases of intramuscular orbital schwannoma.

*Case report*	*Age*	*Gender*	*Muscle involved*	*Surgical technique*
*Capps* et al.*, 1990* (7)	8 years old	Female	Medial rectus	Full thickness biopsy of the muscle
*Kiratli, Yildiz & Söylemezoğlu., 2007* (8)	12 years old	Female	Lateral rectus	Incisional biopsy of the muscle
*Colapinto* et al.*, 2009* (9)	68 years old	Male	Inferior oblique	Excisional biopsy with partial inferior oblique myectomy.
*Jia* et al.*, 2015* (10)	27 years old	Male	Superior oblique	Complete resection en bloc.
*Young* et al.*,2018* (11)	71 years old	Male	Medial rectus	Incisional biopsy of the mass from the muscle.
*Jawada, Coutua & Chiambaretta., 2019* (12)	84 years old	Male	Lateral rectus	No surgical intervention was done given his age and comorbidities. Patient tolerated prisms.

Capps and colleagues in 1990 presented a case of an 8-year-old girl with right-sided proptosis starting three years earlier, as evidenced by previous photos of the girl. Like the case herein, the main presentation was proptosis, 5mm, compared to the other eye. Other ocular examinations were unremarkable. An orbital CT scan shows a mass extending from the insertion of the medial rectus muscle, causing lateral displacement of the optic nerve. On MRI, there was no distinction between the medial rectus muscle and the mass, suggesting that the mass originated from within the muscle. Surgical excision was curative in this case (7).

Kiratli and colleagues 2007 presented a case of a 12-year-old girl with bilateral proptosis upon presentation with a known family history of Neurofibromatosis type 1. Visual acuity was light perception in the right eye and 20/40 in the left eye. MRI showed a cerebellopontine angle tumor, a tumor surrounding the right intraorbital optic nerve. The left eye demonstrated a left lateral orbital mass mixed with the lateral rectus muscle. Biopsy showed an orbital schwannoma in the left eye and nerve sheath meningioma in the right eye (8).

Colapinto and colleagues reported in 2009 a case of a 68-year-old male whose main complaint was reduced vision, hypertropia, limitation of infraduction, and diplopia on down gaze in the right eye. Proptosis was measured to be 3mm compared to the other eye. Exploration and excision were performed through anterior orbitotomy, where the mass could not be distinguished from the inferior oblique muscle. Histological examination confirmed orbital schwannoma. Visual acuity and ocular movement returned to normal after surgery, with no documented recurrence after two years of follow-up (9).

In 2015, Jia and colleagues reported a case of a 27-year-old man with a history of progressive proptosis of the left eye with mild inferior displacement of the eye accompanied by minimal elevation deficiency. MRI showed a well-defined mass in the left superior orbit. Anterior orbitotomy revealed a mass that originated from the superior oblique tendon; complete excision and histology were performed, and the diagnosis of orbital schwannoma was made. Following surgery, all symptoms resolved, and no recurrence was observed during the six years of follow-up (10).

Young and colleagues reported in 2018 a case of a 71-year-old male who presented with left eye visual loss and periorbital inflammation. The main symptom was proptosis of the left eye, which was measured at 6mm compared to the right eye. Visual acuity was 20/20 in the right eye, and vision in the left eye was 20/200, coupled with reduced color vision and a positive relative afferent pupillary defect (APD). On the CT scan, a mass was observed in the left orbit, indenting the globe and displacing it anterolaterally. During surgical excision, the mass was indistinguishable from the medial rectus and is seen arising from it. Following surgical excision, visual acuity returned to 20/25, color vision was restored, and APD resolved. Histology findings were consistent with orbital schwannoma (11).

Jawda and colleagues reported 2019 a case of an 84-year-old male with a medical history of heart disease, high blood pressure, and hyperthyroidism on medical treatment. He underwent an MRI exam due to experiencing debilitating headaches, where an incidental finding of an enlarged left lateral rectus muscle was observed. The mass was found to originate from within the left lateral rectus muscle, not affecting the surrounding structures and respecting the path of the optic nerve. The patient complained of diplopia, which was managed symptomatically by prisms.

A biopsy was performed, and a diagnosis of orbital schwannoma was made. Given the patient’s general health, surgical excision was not justified and thus not performed (12).

Intramuscular orbital schwannoma is a rare entity. Surgical excision is curative with minimal to no effect on surrounding structures or visual function. Only six cases are reported in the literature, with the youngest being an 8-year-old girl. This case is the youngest male reported to have such a lesion.

## Data Availability

The original contributions presented in the study are included in the article/supplementary material. Further inquiries can be directed to the corresponding author.

## References

[1] Brucoli M Giarda M Arcuri F Benech A. A benign isolated schwannoma of the orbit. J Craniofac Surg. (2011) 22:2372. doi: 10.1097/SCS.0b013e318231e4f2, PMID: 22134283

[2] Lam DSC Ng JSK To KF Abdulah V Liew CT Tso MOM. Cystic schwannoma of the orbit. Eye. (1997) 11:798–800. doi: 10.1038/eye.1997.208, PMID: 9537134

[3] Tezer MS Ozcan M Han Ö Unal A Ozlugedik S. Schwannoma originating from the infraorbital nerve: A case report. Auris Nasus Larynx. (2006) 33:343–5. doi: 10.1016/j.anl.2005.11.015, PMID: 16413981

[4] Rootman J Goldberg C Robertson W. Primary orbital schwannomas. Br J Ophthalmol. (1982) 66:194–204. doi: 10.1136/bjo.66.3.194, PMID: 7066273 PMC1039752

[5] Wang Y Xiao LH. Orbital schwannomas: findings from magnetic resonance imaging in 62 cases. Eye. (2008) 22:1034–9. doi: 10.1038/sj.eye.6702832, PMID: 17464300

[6] Guedes-Corrêa JF Cardoso RSV. Immunohistochemical markers for schwannomas, neurofibromas and Malignant peripheral nerve sheath tumors—What can the recent literature tell us? Arquivos Brasileiros Neurocirurgia: Braz Neurosurg. (2018) 37:105–12. doi: 10.1055/s-0038-1667180

[7] Capps DH Brodsky MC Rice CD Mrak RE Glasier CM Brown HH. Orbital intramuscular schwannoma. Am J Ophthalmol. (1990) 110:535–9. doi: 10.1016/S0002-9394(14)77878-X, PMID: 2240140

[8] Kiratli H Yildiz S Söylemezoğlu F. Neurofibromatosis type 2: optic nerve sheath meningioma in one orbit, intramuscular schwannoma in the other. Orbit. (2008) 27:451–4. doi: 10.1080/01676830802350356, PMID: 19085302

[9] Colapinto P Sheth HG Jain R Joshi N Wong T. Inferior oblique schwannoma: diagnosis and management. Orbit. (2007) 26:287–9. doi: 10.1080/01676830601174585, PMID: 18097970

[10] Li J Lin J Liu R Li J Yan J. Orbital schwannoma originating from the superior oblique muscle. J Craniofac Surg. (2015) 26:559. doi: 10.1097/SCS.0000000000001230, PMID: 25643338

[11] Young SM Kim Y-D Hwang SS Woo KI. Orbital schwannoma with atypical presentation. J Craniofac Surg. (2018) 29:e224. doi: 10.1097/SCS.0000000000004177, PMID: 29419597

[12] Jawad V Coutu A Chiambaretta F. Extraocular muscle Schwannoma: A case report. J francais d’ophtalmologie. (2019) 42:e213–5:5. doi: 10.1016/j.jfo.2018.07.020, PMID: 30955901

