# Correction: Intramuscular orbital schwannoma: case report and review of the literature

**DOI:** 10.3389/fopht.2025.1655719

**Published:** 2025-07-17

**Authors:** Hammam A. Alotaibi, Firas Madani, Rawan N. Althaqib, Hamad M. AlSulaiman

**Affiliations:** ^1^ Oculoplastics & Orbit Division, King Khaled Eye Specialist Hospital, Riyadh, Saudi Arabia; ^2^ Department of Ophthalmology, Prince Sultan Military Medical City, Riyadh, Saudi Arabia; ^3^ Department of Ophthalmology, Faculty of Medicine, King Abdulaziz University, Jeddah, Saudi Arabia

**Keywords:** orbital schwannoma, diplopia, magnetic resonance, inferior rectus muscle, proptosis, case report

In the published article, Affiliation “Oculoplastics & Orbit Division, King Khaled Eye Specialist Hospital, Riyadh, Saudi Arabia” was omitted for authors “Hammam A. Alotaibi and Firas Madani”. This affiliation has now been added for authors “Hammam A. Alotaibi^1^, Firas Madani” and accordingly, all the affiliations have been reordered.

“Hammam A. Alotaibi^1,2^, Firas Madani^1,3^, Rawan N. Althaqib^1,^ Hamad M. AlSulaiman^1*^

^1^Oculoplastics & Orbit Division, King Khaled Eye Specialist Hospital, Riyadh, Saudi Arabia

^2^Department of Ophthalmology, Prince Sultan Military Medical City, Riyadh, Saudi Arabia

^3^Department of Ophthalmology, Faculty of Medicine, King Abdulaziz University, Jeddah, Saudi Arabia”

The original version of this article has been updated.

